# Ccn6 Is Required for Mitochondrial Integrity and Skeletal Muscle Function in Zebrafish

**DOI:** 10.3389/fcell.2021.627409

**Published:** 2021-02-11

**Authors:** Archya Sengupta, Deepesh Kumar Padhan, Ananya Ganguly, Malini Sen

**Affiliations:** Division of Cancer Biology & Inflammatory Disorder, CSIR-Indian Institute of Chemical Biology, Kolkata, India

**Keywords:** CCN6, PPRD, muscle, mitochondria, respiratory complex, zebrafish

## Abstract

Mutations in the *CCN6* (*WISP3*) gene are linked with a debilitating musculoskeletal disorder, termed progressive pseudorheumatoid dysplasia (PPRD). Yet, the functional significance of CCN6 in the musculoskeletal system remains unclear. Using zebrafish as a model organism, we demonstrated that zebrafish Ccn6 is present partly as a component of mitochondrial respiratory complexes in the skeletal muscle of zebrafish. Morpholino-mediated depletion of Ccn6 in the skeletal muscle leads to a significant reduction in mitochondrial respiratory complex assembly and activity, which correlates with loss of muscle mitochondrial abundance. These mitochondrial deficiencies are associated with notable architectural and functional anomalies in the zebrafish muscle. Taken together, our results indicate that Ccn6-mediated regulation of mitochondrial respiratory complex assembly/activity and mitochondrial integrity is important for the maintenance of skeletal muscle structure and function in zebrafish. Furthermore, this study suggests that defects related to mitochondrial respiratory complex assembly/activity and integrity could be an underlying cause of muscle weakness and a failed musculoskeletal system in PPRD.

## Introduction

CCN6 (WISP3), a CCN (Cyr61, CTGF, NOV) family member, is a multi-domain protein that is expressed in most cells of mesenchymal origin. Similar to other members of the CCN family, the signal peptide at the N terminus of CCN6 is followed by the insulin growth factor binding protein (IGFBP)-like domain, von Willebrand factor type C (VWR)-like domain, thrombospondin type I (THBS)-like domain, and cysteine knot (CK)-like domain, which have potential for binding to different proteins or peptides and dimerization *via* inter-protein disulfide bond formation (Engel, 2004; Schutze et al., 2005; Holbourn et al., 2008; Katsube et al., 2009; Perbal, 2013). Given the potential of CCN6 to interact with several proteins, it can be expected that loss of CCN6 function resulting from *CCN6* gene mutations or depletion would have diverse effects on cellular functions. In fact, mutations in the *CCN6* gene, which span across the entire length of the protein coding sequence, are linked with a musculoskeletal disorder termed progressive pseudorheumatoid dysplasia (PPRD) (Hurvitz et al., 1999).

Progressive pseudorheumatoid dysplasia, an incurable debilitating disorder, is characterized by loss of cartilage, muscle weakness, and irregular bone growth especially in the joints (Hurvitz et al., 1999; Dalal et al., 2012; Sun et al., 2012; Ekbote et al., 2013; Yang et al., 2013; Liu et al., 2015; Luo et al., 2015; Chouery et al., 2017; Alawbathani et al., 2018; Shahi et al., 2020). But how *CCN6* gene mutations lead to defects in cartilage and muscle remains unclear at the molecular level. In order to gain an in-depth understanding of how PPRD initiates and progresses, one must have a clear picture of the function of the wild-type CCN6 protein and how it interacts with other proteins in different cellular contexts.

Previous reports have demonstrated that CCN6 is present in adult cartilage, mostly in chondrocytes of the mid-zone to the superficial zone and in the fetal growth plate. In chondrocytes, CCN6 maintains the expression of cartilage-specific matrix proteins, such as collagen II and aggrecan, and regulates the production of reactive oxygen species and hypertrophy (Sen et al., 2004; Davis et al., 2006; Miller and Sen, 2007; Repudi et al., 2013). Furthermore, we recently reported that CCN6 localizes to the mitochondria and controls mitochondrial respiratory complex assembly/activity and ATP production (Patra et al., 2016; Padhan et al., 2020). In view of the fact that all the functional assays with reference to CCN6 were performed using cell lines, we wanted to examine the function of CCN6 at the organism level. Accordingly, for a thorough understanding of CCN6 function in the mitochondria and its relation to the musculoskeletal system, we have used zebrafish as our study platform. This choice of organism was prompted by a prior report of a defective cartilage development following morpholino-mediated Ccn6 depletion (Nakamura et al., 2007).

In the current manuscript, we have demonstrated that in the mitochondria of adult zebrafish skeletal muscle, a significant fraction of the total Ccn6 protein is present as a component of the mitochondrial respiratory complexes. Morpholino-mediated depletion of Ccn6 in zebrafish skeletal muscle leads to a considerable reduction in respiratory complex assembly and activity and to a loss of mitochondrial integrity. These mitochondrial defects are associated with abnormal muscle architecture and a diminished muscle function in zebrafish. Our results indicate that defects in the mitochondrial respiratory complex assembly/activity and loss of mitochondrial integrity may constitute an underlying cause of muscle weakness associated with PPRD.

## Materials and Methods

### Animal Maintenance

Adult zebrafish (AB strain) were kept in 30-L fish tanks maintained at 26 ± 2°C temperature in 14:10-h light/dark cycle. All fish tanks were supplied with filtered water. Aeration through an air pump and continuous circulatory flow of water were retained in all the tanks as described in the literature (Avdesh et al., 2012). Fish were fed twice a day with commercially available fish food pellets. Age-matched groups including both males and females were used for experiments. All experiments were performed following internationally approved guidelines (Reed and Jennings, 2010).

### Morpholino Treatment

Zebrafish *ccn6*-specific morpholino oligo was designed and procured from Gene Tools (United States) to block *ccn6* translation. The sequence of the morpholino used is 5′-GTA GTGA TAGCATCA TACACGGCTT-3′. A universal standard control morpholino having the sequence 5′-CCT CTT ACC TCA GTT ACA ATT TAT A-3′ was also procured for use as a negative control (Stainier et al., 2017). Each morpholino oligo was reconstituted in sterile water to make a final concentration of 200 μM for injection into adult fish. For morpholino injection, adult fish of 9–12 months of age were selected. Each fish was either anesthetized with MS222 (0.168 mg/mL, pH 7.5) or subjected to cold shock following internationally approved guidelines (Reed and Jennings, 2010). Subsequently, each fish was injected with 4 μL of morpholino solution at the left dorsal muscle just above the dorsal fin using a 30-gauge Hamilton syringe. After injection, each fish was subjected to electroporation using a tweezer electrode with three pulses of a 50-V current, 50 ms each with 5-s intervals to ensure intracellular delivery of morpholino (Fausett et al., 2008). During injection and electroporation, aerated water was used to irrigate fish gill for avoiding dehydration and ensuring proper respiration. After electroporation, the fish were immediately moved into the water tanks and maintained for ∼65 h at 26 ± 2°C for collection of skeletal muscle following sacrifice. The left dorsal muscle around the injection site was dissected out and labeled as “*ccn6*/Control morpholino injected.” Muscle from the right ventral side of the same fish was also dissected and labeled as “uninjected.”

### Tissue Lysate Preparation and Immunoblotting

Tissue was homogenized with a motorized dounce homogenizer using tissue lysis buffer [50 mM Tris (pH 8.0), 150 mM NaCl, 0.1% SDS, 50 mM DTT, 2 mM PMSF, 1 mM EDTA, 5% glycerol, 5 mM NaF, 2 mM sodium orthovanadate, 0.5% sodium deoxycholate, and 1% Triton-X]. Total protein concentration was measured using Bradford reagent. Following sodium dodecyl sulfate polyacrylamide gel electrophoresis (SDS-PAGE), the proteins were transferred to a polyvinylidene fluoride (PVDF) membrane and kept in a blocking solution [5% bovine serum albumin (BSA) in Tris-buffered saline (TBS) with 0.1% Tween-20] for 2 h. Membranes were then incubated with specific primary antibodies overnight at 4°C [primary antibodies used: zebrafish-specific anti-Ccn6 and anti-Ndufb8 antibody (BioBharati Life Sciences), anti-β-actin antibody (Santa Cruz), anti-Ndufs1 antibody (Thermo Fisher Scientific), anti-Cox-IV antibody (Cell Signaling), anti-Vdac1 antibody (Abcam), anti-Atp5a1 antibody (Thermo Fisher Scientific), and anti-Uqcrc2 antibody (Thermo Fisher Scientific)]. Using appropriate horseradish peroxidase (HRP)-conjugated secondary antibodies, anti-rabbit and anti-mouse IgG (Sigma), the membranes were visualized with a chemiluminescence reagent (Millipore) and the chemiluminescence corresponding to each antibody was documented in a chemi-documentation system.

### Mitochondria Isolation

Mitochondria were isolated from the skeletal muscle tissue of zebrafish by following previously published protocols (Spinazzi et al., 2012; Patra et al., 2016; Padhan et al., 2020). The tissue samples, flash frozen in liquid nitrogen and stored in a -80°C freezer, were thawed on ice. Next, the samples were incubated in isolation buffer 1 [225 mM mannitol, 75 mM sucrose, 0.1 mM EGTA, and 30 mM Tris-HCl (pH 7.4)] and homogenized on ice using a motorized dounce homogenizer for 3 min, followed by 5 min ice incubation. The homogenization and incubation process was repeated three times. The homogenate was then collected and centrifuged at 600 × *g* for 5 min at 4°C. The pellets containing unbroken tissue and nuclei were discarded and the supernatant was further centrifuged at 7,000 × *g* for 10 min to obtain a mitochondria-containing pellet and a cytosolic supernatant. This pellet was washed with isolation buffer 2 [225 mM mannitol, 75 mM sucrose, and 30 mM Tris–HCl (pH 7.4)] and centrifuged at 10,000 × *g* for 10 min at 4°C. The isolated crude mitochondrial pellet was resuspended in mitochondrial resuspension buffer [250 mM mannitol, 0.5 mM EGTA, and 5 mM HEPES (pH 7.4)] and either stored for blue native PAGE (BN-PAGE) analysis or lysed for immunoblotting with mitochondria lysis buffer [50 mM Tris-HCl (pH 7.5), 150 mM NaCl, 0.1 mM EDTA, 1% Triton X-100, and 2 mM 6-amino hexanoic acid]. The protein content was estimated by Bradford reagent.

### Size Exclusion Chromatography

Size exclusion chromatography of endogenous muscle mitochondria was performed using Sephacryl S-300 resin packed with 30 mL bed volume. The column was first washed with buffer containing 50 mM Tris–HCl (pH 8), 150 mM NaCl, and 1 mM EDTA several times and then calibrated with specific molecular weight markers (Sigma). A column calibration curve was prepared using the elute volume of the molecular weight markers and used as a reference scale for each experimental sample elution. Of the mitochondrial lysate, 300 μg was run through the column and fractions were collected (330 μL each). From each fraction, protein was precipitated using 20% TCA and washed with acetone. Dried precipitated samples were then processed for SDS-PAGE and immunoblotting following the protocol described earlier.

### Blue Native PAGE

Frozen mitochondria suspended in resuspension buffer was thawed on ice and estimated by Bradford reagent. About 25 μg of mitochondria was centrifuged at 10,000 × *g* for 10 min at 4°C, after which the pellet was incubated with 20 μl solubilization buffer [50 mM NaCl, 2 mM 6-aminohexanoic acid, 1 mM EDTA, and 50 mM imidazole–HCl (pH 7.0)] and 1 μl dodecyl maltoside (20%) on ice for 10 min. The sample was then subjected to centrifugation for 20 min at 20,000 × *g* at 4°C. The clear supernatant was mixed with 1.5 μL 50% glycerol and 1 μl native loading dye (5% G250 Coomassie Blue in 500 mM 6-aminohexanoic acid) before loading onto a 3–13% gradient gel (Wittig et al., 2006). The gel was run in a cold room for about 8 h at 80 V. After BN-PAGE, the gel was processed for immunoblotting. Briefly, the proteins were transferred to a PVDF membrane for 90 min at 30 V at room temperature using a semi-dry transfer system (Hoefer). Following transfer, the attached Coomassie dye on the membrane was removed by rinsing it with methanol. Finally, the membrane was processed for antibody staining and protein detection following the immunoblotting procedure described earlier.

### Measurement of Complex I Activity

Mitochondrial complex I (NADH dehydrogenase) activity was measured from the isolated mitochondria by following published protocol (Spinazzi et al., 2012; Patra et al., 2016; Padhan et al., 2020), with minor modifications. Briefly, the protein was estimated from freshly isolated mitochondria by the Bradford method. Accordingly, equal amounts of mitochondria from the experimental and control samples were taken and centrifuged at 10,000 × *g* for 10 min at 4°C to obtain mitochondria pellet. The pellet was resuspended in 10 mM ice-cold hypotonic Tris buffer (pH 7.6) and subjected to three cycles of freeze–thawing. Subsequently, the sample was mixed with an assay buffer [50 mM potassium phosphate buffer (pH 7.5), 3 mg/mL BSA, and 3 mM sodium azide], to which 100 μM NADH and 100 μM ubiquinone were added. NADH reduction (extinction coefficient, 6.22 mM^–1^ cm^–1^) was correlated with a decrease in the absorbance of NADH (340 nm) at different time points over a course of 5 min. Complex I activity (nmol min^–1^ mg^–1^) was calculated as: (ΔAbsorbance/min × 1,000)/[(extinction coefficient × volume of sample used in mL) × (sample protein concentration in mg mL^–1^)] (Spinazzi et al., 2012). Percent change of activity was calculated considering activity of the control sample as 100.

### Measurement of Mitochondrial ATP Synthesis

Mitochondrial ATP synthesis was measured by a bioluminescence assay. Briefly, freshly isolated mitochondria (100 μg/mL) from frozen tissue was energized by incubating in a respiration buffer [0.6 M sorbitol, 1 mM MgCl_2_, 1 mM EDTA, 25 mM succinate, 5 mM ADP, and 25 mM potassium phosphate buffer (pH 7.0)] for 15 min at 37°C, based on published literature (Mittal et al., 2009; Patra et al., 2016). The ATP generated was quantified by using an ATP determination kit (Thermo Fisher Scientific) following the manufacturer’s protocol. Luminescence was measured by using HIDEX Sense Multimode Micro Plate Reader 425-301 (HIDEX).

### Histology and Immunofluorescence

**FIGURE 1:**
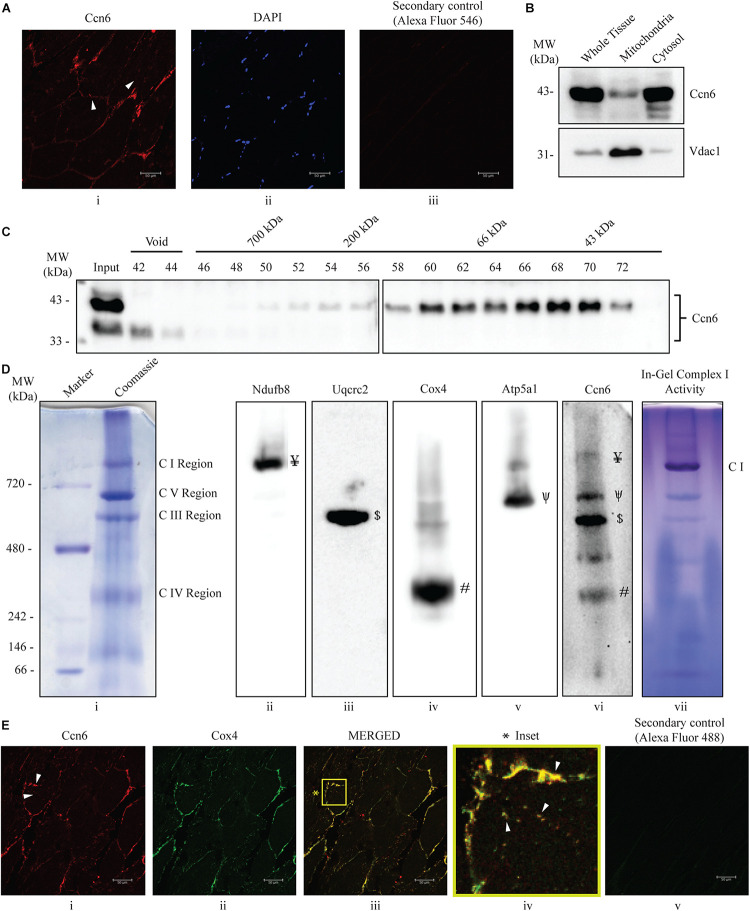
Ccn6 is expressed in zebrafish skeletal muscle and remains associated with mitochondrial respiratory complexes. **(A)** Immunostaining and confocal microscopy demonstrating Ccn6 expression in zebrafish skeletal muscle. **(i)** Ccn6 expression in the fiber lining near the sarcolemma and in patches in inter-myofibril spaces, as shown by *arrows*; **(ii)** DAPI stain showing nuclei; **(iii)** only secondary antibody control. **(B)** Ccn6 expression in skeletal muscle mitochondria in comparison with whole tissue and cytosol. Vdac1 is used as a reference as a mitochondrial protein. **(C)** Sephacryl S-300 gel filtration of zebrafish muscle mitochondrial lysate demonstrating the presence of Ccn6 in high molecular weights encompassing those of mitochondrial respiratory complexes. **(D)** Blue native (BN)-PAGE of muscle mitochondria **(i)** and immunoblotting separately with antibodies to Ndufb8 of complex I **(ii)**, Uqcrc2 of complex III **(iii)**, Cox4 of complex IV **(iv)**, Atp5a1 of complex V **(v)**, and Ccn6 **(vi)**. *Symbols on the blots* **(ii–v)** denote the positions of different complexes corresponding with the Ccn6 bands in panel **(vi)**. In-gel nitro blue tetrazolium chloride (NBT) assay after BN-PAGE of the mitochondria demonstrates the activity of assembled complex I **(vii)**. **(E)** Immunostaining demonstrating the co-localization of Ccn6 with the mitochondrial respiratory complex. **(i)** Ccn6 expression; **(ii)** Cox4, representing mitochondrial respiratory complex IV expression; **(iii)** merged view showing the co-localization of Ccn6 with Cox4; **(iv)** magnified view (*inset*) of the co-localization (*arrowheads*); and **(v)** secondary antibody control for Cox4. The presented data **(A,E)** are representative of three independent experiments. For pooled experiments **(B–D)**, the number of fish used were 3, 25, and 10, respectively.

Skeletal muscle was dissected out and fixed in 10% buffered formalin overnight. The sample was then dehydrated in graded alcohol, embedded in paraffin, sectioned using a microtome at a thickness of 3 μM, and subjected to routine histology and immunofluorescence. For histological study, the sections were mounted in Mayer’s albumin-coated glass slides and stained with hematoxylin–eosin following standard procedure (Feldman and Wolfe, 2014). For immunofluorescence study, the tissue sections were mounted on poly-L-lysine-coated glass slides and processed accordingly following standard protocol (Scanziani, 1998; Joshi and Yu, 2017). The prepared slides were immersed in Tris-EDTA buffer [10 mM Tris base, 1 mM EDTA, 0.05% Tween 20 (pH 9.0)] and placed in a pressure cooker for heat-induced antigen retrieval. The slides were then incubated with primary antibody (anti-Vdac1, anti-Ccn6, or anti-Cox4) overnight at 4°C after blocking with 10% normal serum and 1% BSA in TBS. An appropriate fluorophore-conjugated secondary antibody [Alexa Fluor 546 anti-rabbit antibody or Alexa Fluor 488 anti-mouse antibody (Thermo Fisher Scientific)] was used to detect the signal under a confocal microscope (Figures 1A,E, 3A) and a fluorescence microscope (Supplementary Figure 1).

### Analysis of Swimming Behavior

The swimming behavior of zebrafish after the administration of morpholino was analyzed following published protocol of “startle response” (Eddins et al., 2010; Miller et al., 2012), with slight modifications. After injection, individual fish was kept in a small white rectangular tank (28 cm length × 20 cm width × 10 cm height) with 4 L of system water. After 48 or 72 h post-morpholino injection, a “single tap” on the tank was used to create stimulus. Swimming behavior after tapping was recorded up to 4 min with a digital video camera fixed at the top of the tank. Uninjected fish was also used as a reference. Captured video recordings were analyzed with video analysis software (Filmora9) to determine the distance covered and the turns taken per minute by each fish.

### Statistical Analysis

All the data were presented as the mean ± SEM. Statistical analysis was performed using GraphPad Prism software, and *p* value was calculated by Student’s *t* test. For the evaluation of differences between the experimental and control groups, three to five fish were used for each group for a particular data point. For experiments involving mitochondria, where samples were pooled, each pool represented about 10 fish, and any one data point represented at least three pools. For the analysis of immunoblot band intensity, statistical analysis was done after densitometry using GelQuant.NET software provided by biochemlabsolutions.com.

## Results

### Ccn6 Is Present as a Component of Mitochondrial Respiratory Complexes in Zebrafish Skeletal Muscle

Earlier studies using human cell lines demonstrated that CCN6 is associated with the mitochondria, regulates respiratory complex assembly and activity, and controls ATP production (Patra et al., 2016; Padhan et al., 2020). Since mitochondrial assembly and activity are essential for muscle function (Vincent et al., 2016), we wanted to decipher whether Ccn6 is in any way associated with the respiratory complexes of muscle mitochondria. This study was particularly important for understanding CCN6 function in the context of PPRD, where *CCN6* mutations are associated with muscle fatigue and wasting (Alawbathani et al., 2018; Al Kaissi et al., 2019). Accordingly, we used zebrafish as a model organism for our study.

Initially, we demonstrated that Ccn6 is expressed in zebrafish skeletal muscle both by immunostaining and immunoblotting with an anti-Ccn6 antibody. Mitochondrial distribution of Ccn6 with respect to whole tissue was also evaluated [Figures 1A(i–iii),B]. Subsequently, we observed that Ccn6 is present not only in its native molecular weight form but also in the form of high-molecular-weight complexes in the range of 66–1,000 kDa and beyond in zebrafish skeletal muscle mitochondria. This was demonstrated by size exclusion chromatography of the zebrafish skeletal muscle mitochondrial lysate followed by immunoblotting of the collected fractions with the anti-Ccn6 antibody (Figure 1C). BN-PAGE of the zebrafish muscle mitochondria (Figure 1D, panel i) and immunoblotting using the same anti-Ccn6 antibody validated that, in zebrafish muscle mitochondria, a significant fraction of Ccn6 exists as high-molecular-weight complexes (Figure 1D, panel vi). Additional immunoblotting after BN-PAGE, separately with antibodies against mitochondrial respiratory complex subunits, revealed that the high-molecular-weight fraction of Ccn6 is at least partially present in association with complex I (panel ii: Ndufb8), complex III (panel iii: Uqcrc2), complex IV (panel iv: Cox4), and complex V (panel v: Atp5a1). Antibodies against the NDUFB8 subunit (complex I), UQCRC2 subunit (complex III), COX4 subunit (complex IV), and the ATP5A1 subunit (complex V) have been widely used for verifying mitochondrial respiratory complexes (Wittig et al., 2006; Acín-Pérez et al., 2008; Padhan et al., 2020). Complex I activity, as depicted in panel vii of Figure 1D by in-gel nitro blue tetrazolium chloride (NBT) assay (Padhan et al., 2020) validated complex assembly. The association of Ccn6 with mitochondrial respiratory complexes was furthermore verified by immunostaining, where Ccn6 was found to co-localize with Cox4. The co-localization of Ccn6 and Cox4 along the edges of the muscle fibers and in small patches, as depicted by arrow marks, was indicative of their association with the mitochondria, which are present both in connection with the sarcolemma and in inter-myofibril spaces (Figure 1E, i–iv; Percival et al., 2013; Lee et al., 2016). Given the potential of CCN6 for inter-protein interactions by virtue of its modular architecture (Hurvitz et al., 1999), these results led us to investigate whether Ccn6 stabilizes the assembly and activity of mitochondrial respiratory complexes through chaperone-like activity.

### Ccn6 Depletion in Zebrafish Skeletal Muscle Leads to Diminished Mitochondrial Respiratory Complex Assembly and Activity

Having demonstrated that Ccn6 is associated with mitochondrial respiratory complexes in zebrafish skeletal muscle, we investigated whether Ccn6 controls mitochondrial respiratory complex assembly and activity therein. In this context, the effect of Ccn6 depletion was assessed. Depletion of Ccn6 was obtained by injecting *ccn6*-specific morpholino (4 μL of 200 μM morpholino solution in water) into the dorsal side of zebrafish muscle followed by electroporation. For each morpholino-injected muscle tissue sample excised from the left dorsal side of a fish, an uninjected sample of equal size was excised from the right ventral side as a negative control. A similar procedure was exercised with an equal concentration of a non-targeted morpholino as an additional negative control. All experimental and control samples were collected about 65 h post-electroporation after sacrificing the fish.

**FIGURE 2:**
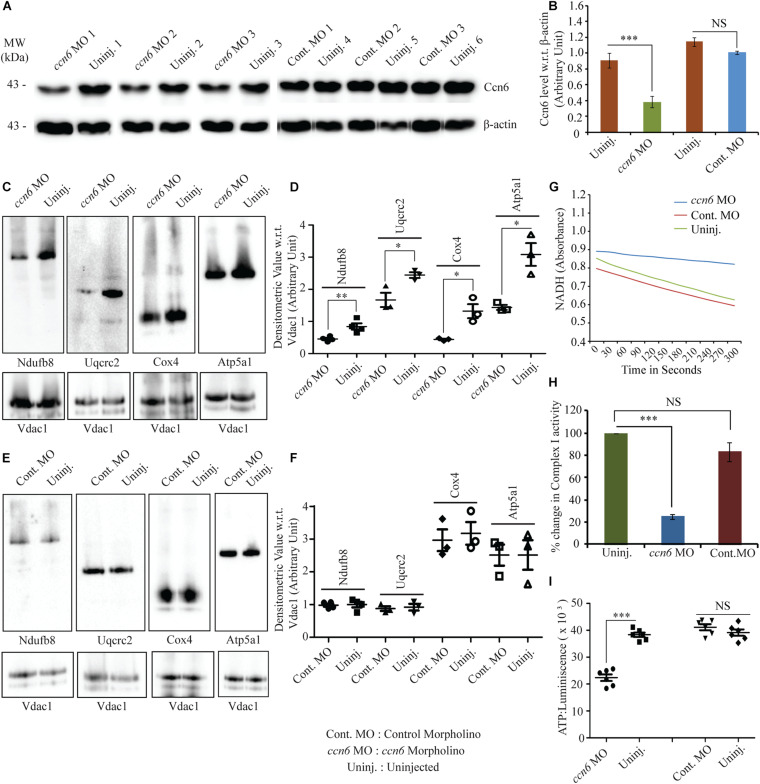
Ccn6 depletion inhibits mitochondrial respiratory complex assembly and activity in zebrafish muscle. **(A,B)** Immunoblotting of the total muscle lysate and corresponding band densitometry demonstrating about 60% reduction in Ccn6 expression by *ccn6* morpholino, but not control morpholino, as compared to the corresponding uninjected control. β-actin is used as a reference for the estimation of Ccn6 expression. **(C–F)** Blue native (BN)-PAGE of the muscle mitochondrial lysate followed by **(i)** immunoblotting separately with antibodies to Ndufb8 (complex I), Uqcrc2 (complex III), Cox4 (complex IV), and Atp5a1 (complex V) and **(ii)** corresponding band densitometry projected by a distribution plot, demonstrating that *ccn6* morpholino, but not non-targeted morpholino, inhibits respiratory complex assembly as compared to no injection. Vdac1 expression from equal amounts of mitochondrial protein is used as a reference for this estimation. **(G,H)** Spectrophotometric and bar graph representation of the decrease in complex I activity upon Ccn6 depletion by *ccn6* morpholino, but not control morpholino. No injection is a reference for this estimation. **(I)** ATP measurement assay demonstrating the decrease in ATP synthesis upon *ccn6* morpholino (MO), but not control MO injection, as compared to the uninjected control. Data are presented as the mean ± SEM of at least three independent experiments. ^∗^*p* < 0.05, ^∗∗^*p* < 0.01, ^∗∗∗^*p* < 0.001; *NS*, not significant (Student’s *t*-test). For pooled experiments **(C–I)**, the number of fish used were 10, 10, 10, and 5, respectively, in each experimental group.

Figure 2A (immunoblotting with Ccn6 antibody) and Figure 2B (corresponding densitometry) depict that an average of about 60% depletion of Ccn6 expression in the skeletal muscle of individual fish was obtained by *ccn6* morpholino, but not the non-targeted (control) morpholino injection, with reference to the uninjected control. Depletion of Ccn6 by morpholino administration was also demonstrated by immunostaining of the skeletal muscle tissue (Supplementary Figure 1).

**FIGURE 3:**
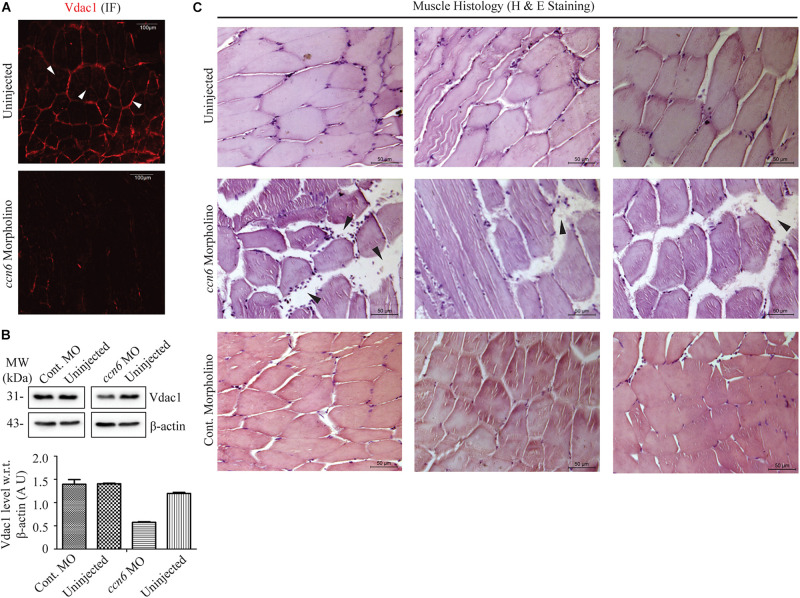
Ccn6 depletion causes loss of muscle mitochondrial abundance and alterations in muscle organization. **(A)** Immunofluorescence of the muscle sections of *ccn6* morpholino-injected and uninjected zebrafish using anti-Vdac1 antibody with Alexa Fluor 546 rabbit secondary antibody, demonstrating loss of Vdac1 (mitochondrial structural protein) expression upon Ccn6 depletion: representation of three different experimental sets. *Arrow marks* denote the presence of mitochondria along the muscle fiber edges near the sarcolemma and in patches in the inter-myofibril spaces. **(B)** Immunoblot of the muscle lysate and corresponding densitometry showing a reduction in Vdac1 levels in *ccn6* morpholino, but not control morpholino-injected fish, as compared to the uninjected control. β-actin is used as a reference protein for this estimation. Data are presented from two independent experiments; tissue was pooled from 10 fish in each experiment. **(C)** Histology (hematoxylin and eosin staining) of the muscle sections from *ccn6* morpholino/control morpholino-injected and uninjected zebrafish demonstrating an increase in the interstitial gaps and presence of inflammatory infiltrates (*arrow marks*) in *ccn6* morpholino-injected muscle: representation of five different experimental sets.

*ccn6*-specific morpholino-mediated depletion of Ccn6 in zebrafish skeletal muscle resulted in a significant reduction in the assembly of complexes I, III, IV, and V in zebrafish muscle mitochondria. Non-targeted (control) morpholino injection did not result in a similar change in the respiratory complex assembly, confirming the specific effect of *ccn6* morpholino on mitochondrial respiratory complexes. This was demonstrated by BN-PAGE of equal amounts of skeletal muscle mitochondria from the experimental (*ccn6* morpholino) and control (non-targeted morpholino and uninjected) samples, followed by immunoblotting separately with antibodies to mitochondrial respiratory complex subunits (complex I: Ndufb8, complex III: Uqcrc2, complex IV: Cox4, and complex V: Atp5a1) and subsequent densitometry. Vdac1 (mitochondrial porin), a mitochondrial structural protein, was used as the reference in the analysis (Figures 2C–F). Defective assembly of the respiratory complexes upon Ccn6 depletion moreover resulted in a significant drop in complex I activity (Figures 2G,H). This finding is in accordance with the already reported involvement of complexes III–V in complex I function (Mimaki et al., 2012; Moreno-Lastres et al., 2012; Milenkovic et al., 2017). The inhibited complex I activity upon Ccn6 depletion was also linked with a marked reduction in mitochondrial ATP synthesis (Figure 2I), indicating a blockade in mitochondrial respiration. The observed changes in the mitochondrial respiratory complex assembly/activity and ATP synthesis upon Ccn6 depletion were associated with the altered distribution of Ccn6 itself among the different respiratory complexes, validating that Ccn6 regulates mitochondrial respiration as a component of the different respiratory complexes. Clearly, *ccn6* morpholino-injected but not control morpholino-injected muscle had overall less mitochondrial Ccn6 protein as well as alteration in its relative distribution among the different mitochondrial respiratory complexes as compared to the uninjected control (Supplementary Figure 2).

### Defective Mitochondrial Respiratory Complex Assembly and Activity in Ccn6-Depleted Skeletal Muscle Dampens Mitochondrial Integrity, Causing Anomalous Muscle Organization and Function

We were interested in investigating how loss of mitochondrial respiratory complex assembly and activity in zebrafish skeletal muscle upon Ccn6 depletion, as described in Figure 2, influences mitochondrial integrity and muscle physiology. This was particularly important on account of the need of a functional mitochondria for proper muscle function (Sunitha et al., 2016; Vincent et al., 2016).

Microtome sections generated from *ccn6* morpholino-injected experimental and matched control zebrafish skeletal muscle samples were prepared for immunofluorescence microscopy with antibody against Vdac1, which, being a mitochondrial structural protein, serves as an indicator of mitochondrial integrity (Padhan et al., 2020). Vdac1 stain was visible both along the edges of the muscle fibers and in patches denoting inter-myofibril spaces, as depicted for Ccn6 and Cox4 in Figure 1. The markedly low level of Vdac1 in the sections prepared from Ccn6-depleted muscle samples as opposed to the controls indicated loss of mitochondrial abundance (Figure 3A). This result was corroborated by immunoblotting muscle lysates obtained from *ccn6* morpholino/control morpholino-injected and uninjected fish with Vdac1 antibody (Figure 3B). As depicted by densitometry with reference to β-actin, while there was about a 50% reduction in Vdac1 level in *ccn6* morpholino-injected muscle as compared to the uninjected muscle, no significant difference was noted between the uninjected and control morpholino-injected muscle samples. Thus, the marked reduction in respiratory complex assembly and activity in zebrafish skeletal muscle upon Ccn6 depletion was associated with loss of mitochondria, indicating a decline in mitochondrial integrity.

In light of the fact that muscle physiology is dependent on mitochondrial function (Vincent et al., 2016), we examined whether the loss of mitochondria in zebrafish skeletal muscle due to Ccn6 depletion correlated with alterations in skeletal muscle structure and function. Accordingly, microtome sections generated from *ccn6* morpholino-injected experimental and matched control zebrafish muscle samples were prepared for histology (hematoxylin and eosin staining). As depicted in Figure 3C, histology revealed that the mitochondrial defects in zebrafish muscle observed upon Ccn6 depletion are associated with a widening of the interstitial spaces between the muscle fibers and inflammatory infiltrates, features which have been linked with muscle wasting (Rayavarapu et al., 2013; Yang et al., 2019). Unlike the *ccn6* morpholino-treated muscle samples, the control morpholino samples appeared more or less like the uninjected samples in the histology. The very minor variations in the interstitial spaces that were observed in some control morpholino samples were perhaps due to injury during electroporation or were just naturally occurring differences in the tissue architecture of different fish. These results confirmed a specific role of Ccn6 in the maintenance of muscle integrity.

**FIGURE 4:**
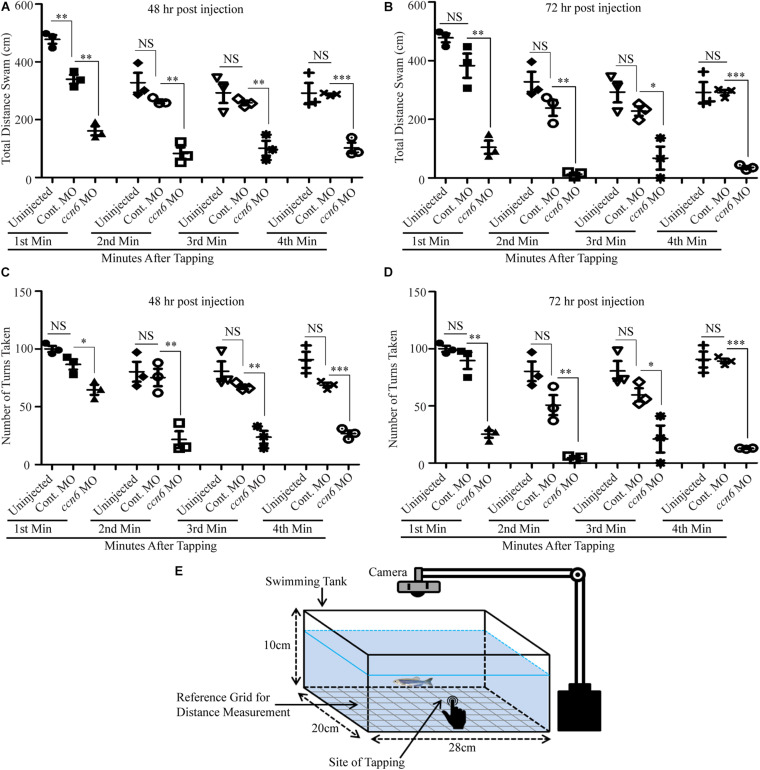
Depletion of Ccn6 expression in zebrafish skeletal muscle inhibits locomotion in response to stimulus. **(A,B)** Ccn6 depletion in skeletal muscle by *ccn6* morpholino restricts the distance covered by zebrafish in response to stimulus as compared to the corresponding controls (control morpholino-injected and uninjected) at 48 and 72 h post-injection (*n* = 3). **(C,D)** Significantly less number of turns by *ccn6* morpholino-injected fish as compared to the corresponding controls at 48 and 72 h post-injection (*n* = 3). Data are presented as the mean ± SEM. ^∗^*p* < 0.05, ^∗∗^*p* < 0.01, ^∗∗∗^*p* < 0.001; *NS*, not significant (Student’s *t* test). **(E)** Representation of the experimental setup.

The altered skeletal muscle structure upon Ccn6 depletion was reflected in the hindered locomotion in the experimental fish as compared to the corresponding controls in response to stimulus. As depicted in Figure 4, *ccn6* morpholino-injected zebrafish, at both 48 and 72 h post-injection, were unable to travel the same distance and perform as many turns as the controls (uninjected and control morpholino-injected zebrafish) after being subjected to a startle, generated by a single tap on the fish tank. The distance covered was measured separately for each fish after the first, second, third, and fourth minutes following tapping (Figures 4A–D). The setup of the “startle response experiment” adapted from contemporary literature (Miller et al., 2012; Lebold et al., 2013) is described in Figure 4E.

Overall, our results indicate that Ccn6 regulates skeletal muscle organization and function through its influence on the mitochondrial respiratory complex assembly and activity.

## Discussion

Based on studies in human chondrocyte lines demonstrating that CCN6 localizes to the mitochondria and regulates mitochondrial respiratory complex assembly and activity, we intended to validate the function of CCN6 at the organism level. Therefore, we characterized Ccn6 with respect to mitochondrial function using zebrafish as a model organism. We found that Ccn6 is present partly as a component of mitochondrial respiratory complexes in zebrafish skeletal muscle tissue and that depletion of Ccn6 in the skeletal muscle damages respiratory complex assembly/activity along with mitochondrial integrity. Moreover, loss of mitochondria due to Ccn6 depletion is associated with defects in skeletal muscle morphology and locomotion in zebrafish.

Prior studies describing the role of CCN6 in mitochondrial function using human chondrocyte lines revealed that about 35% depletion of CCN6 elevates mitochondrial mass and raises mitochondrial respiratory complex assembly and activity (Patra et al., 2016; Padhan et al., 2020). This result may seem contradictory to the current finding where about 60% Ccn6 depletion in skeletal muscle tissue leads to loss in mitochondrial abundance as well as respiratory complex assembly and activity. It must be noted, however, that on account of the potential of CCN6 to interact with multiple proteins, its regulatory influence on the mitochondria may include both repression and activation, depending on its protein interacting partners, which may vary between tissue and cell types. Thus, the extent of CCN6 depletion may have different effects on the mitochondria. While about 35% depletion of CCN6 in cartilage chondrocytes may abolish the formation of CCN6-associated repressor complexes, leading to an overall increase in mitochondrial function (Patra et al., 2016; Padhan et al., 2020), more than 60% depletion of Ccn6 in zebrafish muscle tissue may very likely produce a more global effect on Ccn6 activities, causing mitochondrial dysfunction. Accordingly, *CCN6* mutations that are similar to those associated with PPRD lead to loss of mitochondrial activity in chondrocytes, possibly on account of a totally collapsed CCN6 function (Padhan et al., 2020).

Since most tissues and cell types need a functional mitochondria for survival, one may wonder why PPRD-related defects due to *CCN6* mutations are manifested only in skeletal tissues (Hurvitz et al., 1999; Dalal et al., 2012; Garcia Segarra et al., 2012; Sun et al., 2012; Ekbote et al., 2013; Yang et al., 2013; Liu et al., 2015; Luo et al., 2015; Shahi et al., 2020). Perhaps the mitochondrial distribution pattern of Ccn6 varies among the different cell and tissue types, and accordingly, the influence of *ccn6* mutations is more marked in skeletal tissues as compared to others. From Supplementary Figure 3, it is in fact clear that the Ccn6 distribution patterns in the mitochondria of the skeletal muscle, brain, and heart of zebrafish are distinctly different. Supplementary Figure 3A demonstrates the relative levels of the Ccn6 protein in the mitochondria of the muscle, brain, and heart in relation to Vdac1 (mitochondrial structural protein) and subunits of the mitochondrial respiratory complexes (Ndufb8, Ndufs1: complex I; Cox4: complex IV). It appears that the levels of Ccn6 are different in the mitochondria of these tissues, with muscle containing the most. Also importantly, the mitochondria of these tissues have different forms of Ccn6. While the muscle mitochondria harbor mostly a 43-kDa form of Ccn6, the heart mitochondria harbor mostly a 33-kDa form. The brain mitochondria, on the other hand, harbor almost equal amounts of both forms. Although not clearly understood how, Ccn6 distribution among the different respiratory complexes is clearly more prominent in zebrafish skeletal muscle as compared to the brain and heart (Supplementary Figure 3B). Ccn6 protein distribution in the mitochondria of other skeletal tissues could be the same as in skeletal muscle, thus rendering the skeletal system more susceptible to the deleterious effects of *ccn6* mutations. It is not to be ruled out, however, that in view of the distribution of Ccn6 in subcellular organelles other than the mitochondria (Figure 1B), skeletal muscle damage due to Ccn6 mutations or depletion may also occur independent of the mitochondria.

Taken together, our study on zebrafish reveals a crucial role of Ccn6 in mitochondrial respiration and the maintenance of muscle integrity. In light of the fact that *CCN6* mutations cause muscle weakness in PPRD (Hurvitz et al., 1999; Dalal et al., 2012; Garcia Segarra et al., 2012; Sun et al., 2012; Ekbote et al., 2013; Yang et al., 2013; Liu et al., 2015; Luo et al., 2015; Alawbathani et al., 2018; Shahi et al., 2020), our findings open up a new theme in the study of CCN6 in the context of PPRD pathogenesis. The results garnered from this new direction of research may lead to significant progress in the diagnosis and understanding of PPRD and similar musculoskeletal disorders.

## Data Availability Statement

The raw data supporting the conclusions of this article will be made available by the authors, without undue reservation.

## Ethics Statement

Ethical review and approval was not required for the animal study because Zebrafish does not fall under the purview of the Committee for the Purpose of Control and Supervision of Experiments on Animals (CPCSEA), Govt. of India. Nevertheless, all experiments were performed following internationally approved guidelines.

## Author Contributions

MS conceptualized the research, analyzed the data, and wrote the manuscript. AS performed research, analyzed data, and assisted in writing the manuscript. DP performed research, analyzed data, and assisted in writing the manuscript. AG provided expert technical assistance. All authors contributed to the article and approved the submitted version of the manuscript.

## Conflict of Interest

The authors declare that the research was conducted in the absence of any commercial or financial relationships that could be construed as a potential conflict of interest.

## References

[B1] Acín-Pérez R. Fernández-Silva P. Peleato M. L. Pérez-Martos A. Enriquez J. A. (2008). Respiratory active mitochondrial supercomplexes. *Mol. Cell* 32 529–539. 10.1016/j.molcel.2008.10.021 19026783

[B2] Al Kaissi A. Kenis V. Jemaa L. B. Sassi H. Shboul M. Grill F. (2019). Skeletal phenotype/genotype in progressive pseudorheumatoid chondrodysplasia. *Clin. Rheumatol.* 39 553–560. 10.1007/s10067-019-04783-z 31628567

[B3] Alawbathani S. Kawalia A. Karakaya M. Altmüller J. Nürnberg P. Cirak S. (2018). Late diagnosis of a truncating WISP3 mutation entails a severe phenotype of progressive pseudorheumatoid dysplasia. *Cold Spring Harb. Mol. Case Stud.* 4 a002139. 10.1101/mcs.a002139 29258992 PMC5793772

[B4] Avdesh A. Chen M. Martin-Iverson M. T. Mondal A. Ong D. Rainey-Smith S. (2012). Regular care and maintenance of a zebrafish (*Danio rerio*) laboratory: an introduction. *J. Vis. Exp.* 69 e4196. 10.3791/4196 23183629 PMC3916945

[B5] Chouery E. Corbani S. Dahmen J. Zouari L. Gribaa M. Leban N. (2017). Progressive pseudorheumatoid dysplasia in North and West Africa: Clinical description in ten patients with mutations of WISP3. *Egypt. J. Med. Hum. Genet.* 18 299–303. 10.1016/j.ejmhg.2016.11.004

[B6] Dalal A. Bhavani G. S. L. Togarrati P. P. Bierhals T. Nandineni M. R. Danda S. (2012). Analysis of the WISP3 gene in Indian families with progressive pseudorheumatoid dysplasia. *Am. J. Med. Genet. A* 158A 2820–2828. 10.1002/ajmg.a.35620 22987568

[B7] Davis L. Chen Y. Sen M. (2006). WISP-3 functions as a ligand and promotes superoxide dismutase activity. *Biochem. Biophys. Res. Commun.* 342 259–265. 10.1016/j.bbrc.2006.01.132 16480948

[B8] Eddins D. Cerutti D. Williams P. Linney E. Levin E. D. (2010). Zebrafish provide a sensitive model of persisting neurobehavioral effects of developmental chlorpyrifos exposure: comparison with nicotine and pilocarpine effects and relationship to dopamine deficits. *Neurotoxicol. Teratol.* 32 99–108. 10.1016/j.ntt.2009.02.005 19268529 PMC2885770

[B9] Ekbote A. V. Danda D. Kumar S. Danda S. Madhuri V. Gibikote S. (2013). A descriptive analysis of 14 cases of progressive-psuedorheumatoid-arthropathy of childhood from south India: review of literature in comparison with juvenile idiopathic arthritis. *Semin. Arthritis Rheum.* 42 582–589. 10.1016/j.semarthrit.2012.09.001 23270760

[B10] Engel J. (2004). Role of oligomerization domains in thrombospondins and other extracellular matrix proteins. *Int. J. Biochem. Cell Biol.* 36 997–1004. 10.1016/j.biocel.2003.12.009 15094115

[B11] Fausett B. V. Gumerson J. D. Goldman D. (2008). The proneural basic helix-loop-helix gene Ascl1a is required for retina regeneration. *J. Neurosci.* 28 1109–1117. 10.1523/JNEUROSCI.4853-07.2008 18234889 PMC2800945

[B12] Feldman A. T. Wolfe D. (2014). “Tissue processing and hematoxylin and eosin staining,” in *Histopathology: Methods and Protocols Methods in Molecular Biology*, ed. Day C. E. (New York, NY: Springer), 31–43. 10.1007/978-1-4939-1050-2_3 25015141

[B13] Garcia Segarra N. Mittaz L. Campos-Xavier A. B. Bartels C. F. Tuysuz B. Alanay Y. (2012). The diagnostic challenge of progressive pseudorheumatoid dysplasia (PPRD): a review of clinical features, radiographic features, and WISP3 mutations in 63 affected individuals. *Am. J. Med. Genet. C Semin. Med. Genet.* 160C 217–229. 10.1002/ajmg.c.31333 22791401

[B14] Holbourn K. P. Acharya K. R. Perbal B. (2008). The CCN family of proteins: structure-function relationships. *Trends Biochem. Sci.* 33 461–473. 10.1016/j.tibs.2008.07.006 18789696 PMC2683937

[B15] Hurvitz J. R. Suwairi W. M. Van Hul W. El-Shanti H. Superti-Furga A. Roudier J. (1999). Mutations in the CCN gene family member WISP3 cause progressive pseudorheumatoid dysplasia. *Nat. Genet.* 23 94–98. 10.1038/12699 10471507

[B16] Joshi S. Yu D. (2017). “Chapter 8 – immunofluorescence,” in *Basic Science Methods for Clinical Researchers*, eds Jalali M. Saldanha F. Y. L. Jalali M. (Boston: Academic Press), 135–150. 10.1016/B978-0-12-803077-6.00008-4

[B17] Katsube K. Sakamoto K. Tamamura Y. Yamaguchi A. (2009). Role of CCN, a vertebrate specific gene family, in development. *Dev. Growth Differ.* 51 55–67. 10.1111/j.1440-169X.2009.01077.x 19128405

[B18] Lebold K. M. Löhr C. V. Barton C. L. Miller G. W. Labut E. M. Tanguay R. L. (2013). Chronic vitamin E deficiency promotes vitamin C deficiency in zebrafish leading to degenerative myopathy and impaired swimming behavior. *Comp. Biochem. Physiol. C* 157 382–389. 10.1016/j.cbpc.2013.03.007 23570751 PMC3653440

[B19] Lee H. Kim S.-H. Lee J.-S. Yang Y.-H. Nam J.-M. Kim B.-W. (2016). Mitochondrial oxidative phosphorylation complexes exist in the sarcolemma of skeletal muscle. *BMB Rep.* 49 116–121. 10.5483/bmbrep.2016.49.2.232 26645635 PMC4915115

[B20] Liu L. Li N. Zhao Z. Li W. Xia W. (2015). Novel WISP3 mutations causing spondyloepiphyseal dysplasia tarda with progressive arthropathy in two unrelated Chinese families. *Joint Bone Spine* 82 125–128. 10.1016/j.jbspin.2014.10.005 25553839

[B21] Luo H. Shi C. Mao C. Jiang C. Bao D. Guo J. (2015). A novel compound WISP3 mutation in a Chinese family with progressive pseudorheumatoid dysplasia. *Gene* 564 35–38. 10.1016/j.gene.2015.03.029 25794430

[B22] Milenkovic D. Blaza J. N. Larsson N.-G. Hirst J. (2017). The enigma of the respiratory chain supercomplex. *Cell Metab.* 25 765–776. 10.1016/j.cmet.2017.03.009 28380371

[B23] Miller D. S. Sen M. (2007). Potential role of WISP3 (CCN6) in regulating the accumulation of reactive oxygen species. *Biochem. Biophys. Res. Commun.* 355 156–161. 10.1016/j.bbrc.2007.01.114 17286957

[B24] Miller G. W. Labut E. M. Lebold K. M. Floeter A. Tanguay R. L. Traber M. G. (2012). Zebrafish (*Danio rerio*) fed vitamin E-deficient diets produce embryos with increased morphologic abnormalities and mortality. *J. Nutr. Biochem.* 23 478–486. 10.1016/j.jnutbio.2011.02.002 21684137 PMC3179832

[B25] Mimaki M. Wang X. McKenzie M. Thorburn D. R. Ryan M. T. (2012). Understanding mitochondrial complex I assembly in health and disease. *Biochim. Biophys. Acta* 1817 851–862. 10.1016/j.bbabio.2011.08.010 21924235

[B26] Mittal N. Babu M. M. Roy N. (2009). The efficiency of mitochondrial electron transport chain is increased in the long-lived mrg19 Saccharomyces cerevisiae. *Aging Cell* 8 643–653. 10.1111/j.1474-9726.2009.00518.x 19732042

[B27] Moreno-Lastres D. Fontanesi F. García-Consuegra I. Martín M. A. Arenas J. Barrientos A. (2012). Mitochondrial complex I plays an essential role in human respirasome assembly. *Cell Metab.* 15 324–335. 10.1016/j.cmet.2012.01.015 22342700 PMC3318979

[B28] Nakamura Y. Weidinger G. Liang J. O. Aquilina-Beck A. Tamai K. Moon R. T. (2007). The CCN family member Wisp3, mutant in progressive pseudorheumatoid dysplasia, modulates BMP and Wnt signaling. *J. Clin. Invest.* 117 3075–3086. 10.1172/JCI32001 17823661 PMC1964511

[B29] Padhan D. K. Sengupta A. Patra M. Ganguly A. Mahata S. K. Sen M. (2020). CCN6 regulates mitochondrial respiratory complex assembly and activity. *FASEB J.* 34 12163–12176. 10.1096/fj.202000405RR 32686858

[B30] Patra M. Mahata S. K. Padhan D. K. Sen M. (2016). CCN6 regulates mitochondrial function. *J. Cell Sci.* 129 2841–2851. 10.1242/jcs.186247 27252383

[B31] Perbal B. (2013). CCN proteins: a centralized communication network. *J. Cell Commun. Signal.* 7 169–177. 10.1007/s12079-013-0193-7 23420091 PMC3709049

[B32] Percival J. M. Siegel M. P. Knowels G. Marcinek D. J. (2013). Defects in mitochondrial localization and ATP synthesis in the mdx mouse model of Duchenne muscular dystrophy are not alleviated by PDE5 inhibition. *Hum. Mol. Genet.* 22 153–167. 10.1093/hmg/dds415 23049075 PMC3522404

[B33] Rayavarapu S. Coley W. Kinder T. B. Nagaraju K. (2013). Idiopathic inflammatory myopathies: pathogenic mechanisms of muscle weakness. *Skelet. Muscle* 3 13. 10.1186/2044-5040-3-13 23758833 PMC3681571

[B34] Reed B. Jennings M. (2010). *Guidance on the Housing and Care of Zebrafish (Danio rerio).* London: Royal Society for the Prevention of Cruelty to Animals (RSPCA).

[B35] Repudi S. R. Patra M. Sen M. (2013). WISP3-IGF1 interaction regulates chondrocyte hypertrophy. *J. Cell Sci.* 126 1650–1658. 10.1242/jcs.119859 23424195

[B36] Scanziani E. (1998). Immunohistochemical staining of fixed tissues. *Methods Mol. Biol.* 104 133–140. 9711649 10.1385/0-89603-525-5:133

[B37] Schutze N. Noth U. Schneidereit J. Hendrich C. Jakob F. (2005). Differential expression of CCN-family members in primary human bone marrow-derived mesenchymal stem cells during osteogenic, chondrogenic and adipogenic differentiation. *Cell Commun. Signal.* 3 5. 10.1186/1478-811X-3-5 15773998 PMC1079906

[B38] Sen M. Cheng Y.-H. Goldring M. B. Lotz M. K. Carson D. A. (2004). WISP3-dependent regulation of type II collagen and aggrecan production in chondrocytes. *Arthritis Rheum.* 50 488–497. 10.1002/art.20005 14872491

[B39] Shahi P. Sehgal A. Sudan A. Sehgal S. (2020). Delayed-onset progressive pseudorheumatoid dysplasia with secondary synovial chondromatosis. *BMJ Case Rep.* 13 e234461. 10.1136/bcr-2020-234461 32430353 PMC7239527

[B40] Spinazzi M. Casarin A. Pertegato V. Salviati L. Angelini C. (2012). Assessment of mitochondrial respiratory chain enzymatic activities on tissues and cultured cells. *Nat. Protoc.* 7 1235–1246. 10.1038/nprot.2012.058 22653162

[B41] Stainier D. Y. R. Raz E. Lawson N. D. Ekker S. C. Burdine R. D. Eisen J. S. (2017). Guidelines for morpholino use in zebrafish. *PLoS Genet.* 13:e1007000. 10.1371/journal.pgen.1007000 29049395 PMC5648102

[B42] Sun J. Xia W. He S. Zhao Z. Nie M. Li M. (2012). Novel and recurrent mutations of WISP3 in two Chinese families with progressive pseudorheumatoid dysplasia. *PLoS One* 7:e38643. 10.1371/journal.pone.0038643 22685593 PMC3369844

[B43] Sunitha B. Gayathri N. Kumar M. Keshava Prasad T. S. Nalini A. Padmanabhan B. (2016). Muscle biopsies from human muscle diseases with myopathic pathology reveal common alterations in mitochondrial function. *J. Neurochem.* 138 174–191. 10.1111/jnc.13626 27015874

[B44] Vincent A. E. Ng Y. S. White K. Davey T. Mannella C. Falkous G. (2016). The spectrum of mitochondrial ultrastructural defects in mitochondrial myopathy. *Sci. Rep.* 6 30610. 10.1038/srep30610 27506553 PMC4978969

[B45] Wittig I. Braun H.-P. Schägger H. (2006). Blue native PAGE. *Nat. Protoc.* 1 418–428. 10.1038/nprot.2006.62 17406264

[B46] Yang Q. Yan C. Wang X. Gong Z. (2019). Leptin induces muscle wasting in a zebrafish kras-driven hepatocellular carcinoma (HCC) model. *Dis. Model. Mech.* 12 dmm038240. 10.1242/dmm.038240 30718259 PMC6398506

[B47] Yang X. Song Y. Kong Q. (2013). Diagnosis and surgical treatment of progressive pseudorheumatoid dysplasia in an adult with severe spinal disorders and polyarthropathy. *Joint Bone Spine* 80 650–652. 10.1016/j.jbspin.2013.03.006 23618803

