# Supplementary material for: Ccn6 Is Required for Mitochondrial Integrity and Skeletal Muscle Function in Zebrafish
Source: Front Cell Dev Biol. 2021 Feb 11;9:627409. doi: 10.3389/fcell.2021.627409 (PMC7905066; doi:10.3389/fcell.2021.627409)
Supplement: Supplementary file 1 [file Image_1.pdf]

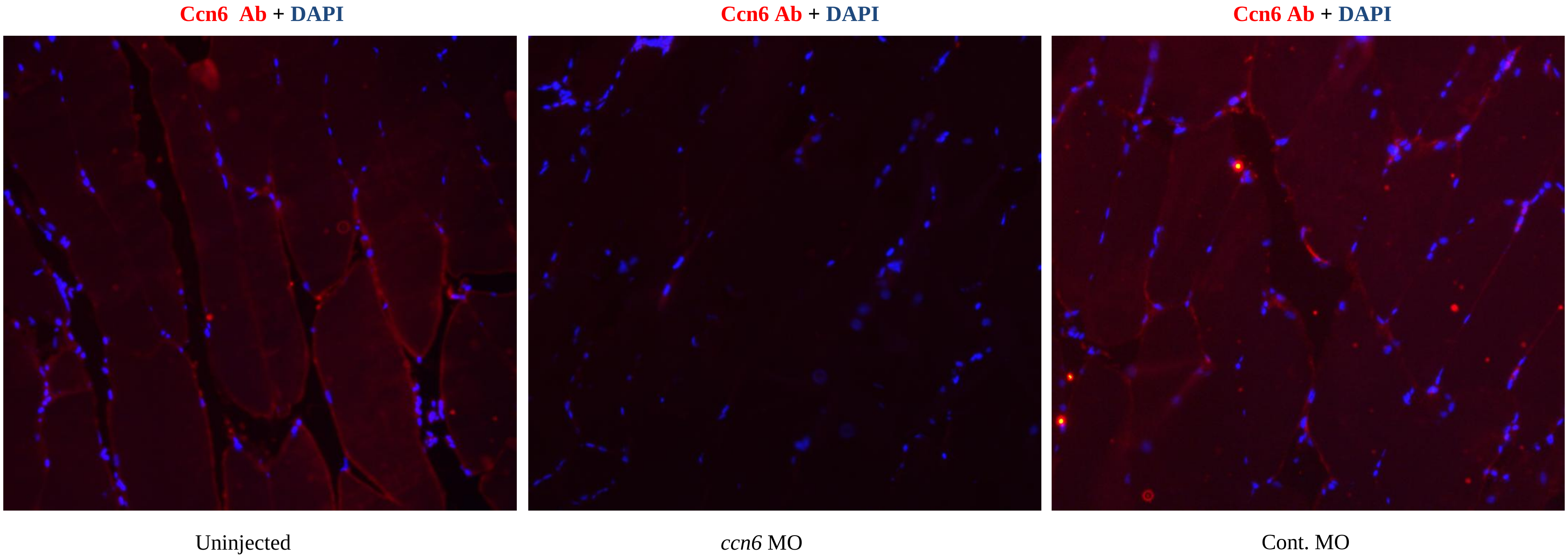

Supplementary Figure 1. Morpholino mediated Ccn6 depletion in skeletal muscle of zebrafish. Fluorescence microscopy of muscle sections stained with anti-Ccn6 antibody (Ab) and counterstained with DAPI showing significant reduction of Ccn6 expression by *ccn6* morpholino administration as compared to control morpholino administration or no administration.

Uninjected : No administration
ccn6 MO : *ccn6* Morpholino adminstration
Cont. MO : Control Morpholino adminstration
Ccn6 Ab : Red (Alexa fluor 546)
DAPI : Blue

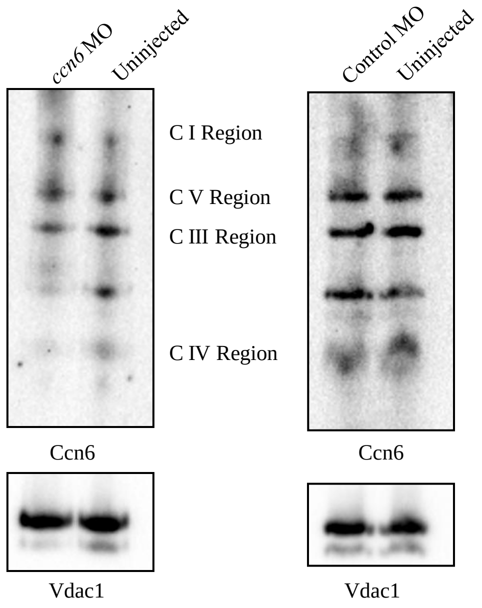

Supplementary Figure 2. Ccn6 depletion in skeletal muscle of zebrafish alters its distribution among mitochondrial respiratory complexes. BN-PAGE of zebrafish muscle mitochondrial lysate and subsequent immunoblotting with Ccn6 antibody demonstrates less Ccn6 protein as well as alteration in its distribution among the respiratory complexes in the muscle mitochondria of *ccn6* morpholino injected but not control morpholino injected fish as compared to the corresponding uninjected controls.

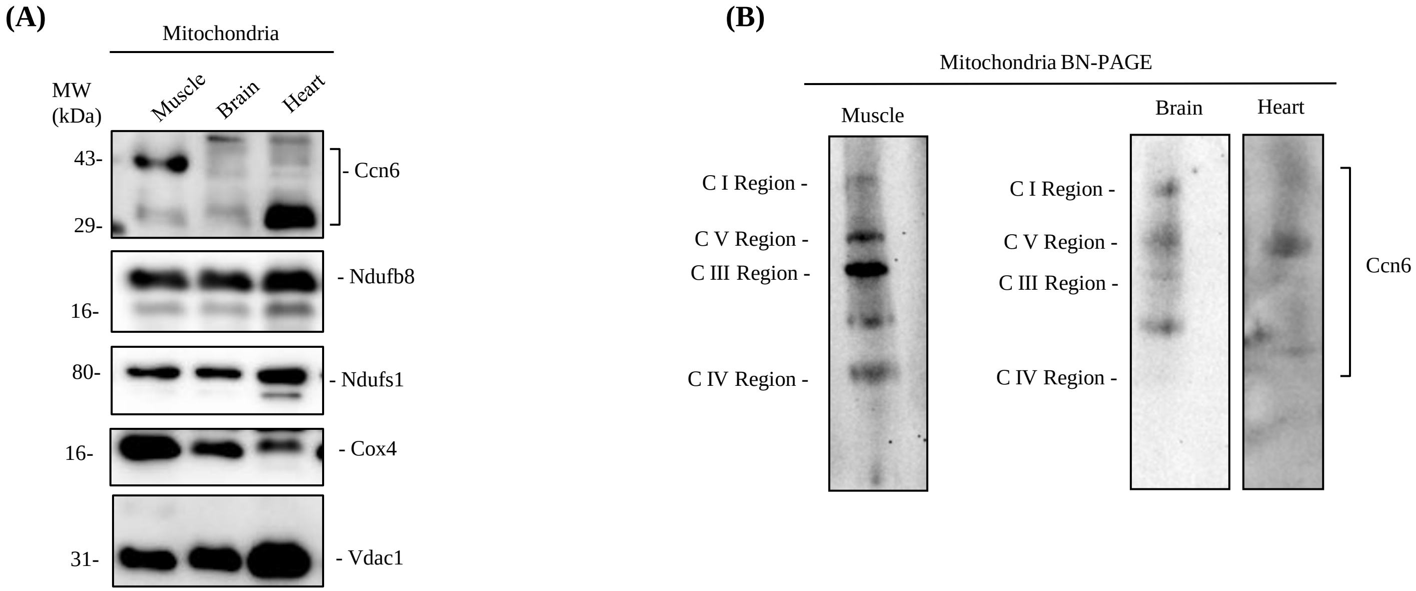

Supplementary Figure 3. Distribution pattern of Ccn6 in mitochondrial respiratory complexes of skeletal muscle is different from that in other tissues. **(A)** Immunoblot of zebrafish mitochondrial lysate with appropriate antibodies showing level of expression of Ccn6 (43kDa and 33kDa) in the mitochondria of muscle, brain and heart of zebrafish with respect to Ndufb8 and Ndufs1 (Complex I subunits), Cox4 (Complex IV subunit) and Vdac1 (porin, a mitochondrial structural protein). **(B)** BN-PAGE and subsequent immunoblots of mitochondrial lysates of muscle, brain and heart of zebrafish with anti-Ccn6 antibody showing different distribution of Ccn6 in the mitochondrial respiratory complexes of muscle, brain and heart.
